# Fatal Neurotoxicosis in Dogs Associated with Tychoplanktic, Anatoxin-a Producing *Tychonema* sp. in Mesotrophic Lake Tegel, Berlin

**DOI:** 10.3390/toxins10020060

**Published:** 2018-01-31

**Authors:** Jutta Fastner, Camilla Beulker, Britta Geiser, Anja Hoffmann, Roswitha Kröger, Kinga Teske, Judith Hoppe, Lars Mundhenk, Hartmud Neurath, Daniel Sagebiel, Ingrid Chorus

**Affiliations:** 1 German Environment Agency, Corrensplatz 1, 14195 Berlin, Germany; ingrid.chorus@uba.de; 2 Berlin Brandenburg State Laboratory Abt. IV, Invalidenstr. 60, 10557 Berlin, Germany; Camilla.Beulker@Landeslabor-BBB.de (C.B.); Anja.Hoffmann@Landeslabor-BBB.de (A.H.); 3 Nusshäherstr. 17A, 13505 Berlin, Germany; geiser.britta@gmail.com; 4 State Office for Health & Social Affairs (LAGeSo), Working Group Water Hygiene & Environmental Health, Postfach 31-09-29, 10639 Berlin, Germany; Roswitha.Kroeger@lageso.Berlin.de (R.K.); Daniel.Sagebiel@lageso.berlin.de (D.S.); 5 Department of Veterinary Pathology, Faculty of Veterinary Medicine, Freie Universität Berlin, Robert-von-Ostertag-Strasse 15, 14163 Berlin, Germany; kinga.teske@fu-berlin.de (K.T.); judith.hoppe@fu-berlin.de (J.H.); Lars.Mundhenk@fu-berlin.de (L.M.); 6 Toxicological Laboratory, Medical University Center, 37075 Göttingen, Germany; hneurath@giz-nord.de

**Keywords:** cyanobacteria, anatoxin-a, *Tychonema*, neurotoxicosis, cyanotoxins, macrophytes

## Abstract

In May 2017, at least 12 dogs showed signs of acute neurotoxicosis after swimming in or drinking from Lake Tegel, a mesotrophic lake in Berlin, Germany, and several of the affected dogs died shortly afterwards despite intensive veterinary treatment. Cyanobacterial blooms were not visible at the water surface or the shorelines. However, detached and floating water moss (*Fontinalis antipyretica*) with high amounts of *Tychonema* sp., a potential anatoxin-a (ATX) producing cyanobacterium, was found near the beaches where the dogs had been swimming and playing. Necropsies of two of the dogs revealed no specific lesions beside the anamnestic neurotoxicosis. ATX was detected in concentrations up to 8700 µg L^−1^ in the stomach contents, while other (neuro)toxic substances were not found. In the aqueous fraction of *Fontinalis*/*Tychonema* clumps sampled after the casualties, ATX was found in concentrations up to 1870 µg L^−1^. This is the first report of a dense population of *Tychonema* sp. in stands of *Fontinalis* resulting in high ATX contents. This case emphasizes the need for further investigation of potentially toxic, non-bloom forming cyanobacteria in less eutrophic water bodies and underlines the novel challenge of developing appropriate surveillance schemes for respective bathing sites.

## 1. Introduction

Hazards due to cyanobacteria are primarily associated with potentially toxic planktic taxa such as *Microcystis*, *Planktothrix*, *Aphanizomenon* and *Dolichospermum*. The potent toxins they may produce comprise hepatotoxins (microcystins), cytotoxins (cylindrospermopsins) and neurotoxins (anatoxins and paralytic shellfish poisons) [1]. Many of these planktic cyanobacteria have the ability to regulate their buoyancy, leading to visible surface blooms and accumulations at shorelines with microcystin (MC) concentrations in the milligram range and thus potentially orders of magnitude higher than in the open water [2,3]. Such blooms may pose a threat to bathers and their surveillance is required according to the European Bathing Water Directive [4]. As toxigenic strains are increasingly identified from benthic taxa such as *Nostoc*, *Oscillatoria*, *Phormidium* and *Lyngbya* [5], the surveillance of benthic cyanobacteria is requested by some countries such as New Zealand [6], but so far not by the European Bathing Water Directive.

Benthic taxa have been found to produce the same range of toxins as planktic cyanobacteria [5,7,8], though most animal fatalities reported in relation to benthic cyanobacteria were associated with the presence of ATX and/or homoanatoxin-a (HATX) [9,10,11,12,13]. Since benthic cyanobacteria attach to surfaces on the bottom of lakes, they are often less apparent than surface blooms. However, benthic mats can detach and accumulate at shorelines [5,14]. There, they may pose a threat to bathers and animals, where among the latter, dogs in particular seem to be attracted to benthic cyanobacteria [15].

While mass developments of planktic cyanobacteria become more prevalent with increasing phosphorus concentrations [16], benthic mats are more independent of water quality due to the influence of benthic microhabitats. Correspondingly, they have been observed over a wide trophic range, even in oligotrophic water bodies [5].

Diverse methods aiming to reduce cyanobacterial abundance in water bodies have been developed. The most sustainable method is a strong reduction of nutrient concentrations, especially of phosphorus, although this is not feasible under all circumstances [17]. One example of success is Lake Tegel, Berlin, Germany, which exhibited mass developments of planktic cyanobacteria during the summer before restoration started in 1984 [18]. Drastic nutrient reduction in the main inflow led to lake recovery after a long transitional period [19,20]. Lake Tegel is now mesotrophic and planktic cyanobacteria, including potentially toxic *Microcystis* and *Dolichospermum*, constituted only low biovolumes of <0.1 mm^3^ L^−1^ between 2014 and 2016 (Beulker and Hoffmann, unpubl. data). Instead, macrophytes have become increasingly abundant in the lake [21].

In May 2017, at least 12 dogs showed a sudden onset of neurological symptoms shortly after swimming in or drinking from Lake Tegel and several dogs died, despite intensive veterinary treatment. No obvious planktic cyanobacterial blooms were visible at the water surface or near the beaches, where the dogs had been. Instead, detached clumps of common water moss (*Fontinalis antipyretica*) were floating near the lakeside, and microscopy revealed high abundance of *Tychonema* sp. within the water moss clumps. Water samples, *Fontinalis* clumps, as well as the stomach contents of three dogs were analyzed for microcystins (MC), cylindrospermopsin (CYN), anatoxin-a (ATX) and homoanatoxin-a (HATX) as well as analogues of ATX and HATX by liquid chromatography tandem mass spectrometry (LC-MS/MS). The stomach contents of the dogs were also investigated for various other (neuro)toxins by gas chromatography mass spectrometry (GC-MS).

## 2. Results

### 2.1. Animal Symptoms and Pathology

Case history data and symptoms of the investigated dogs are summarized in Table 1. The two dogs (D1, D3) submitted for post mortem examination were in a good nutritional status. Several tissues including thymus (dogs D1 and D3), lung, epicard, subcutis, meninges (dog D1) and pancreas (dog D3) showed acute, multifocal hemorrhages. The stomachs of both dogs were well-filled with partially digested food of a moldy-fishy odor and with no further relevant content.

**Figure 1:**
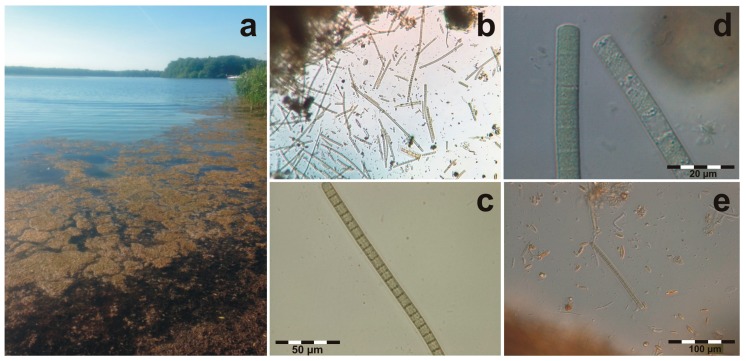
(**a**) Water moss (*Fontinalis antipyretica*) floating on the surface of Lake Tegel (2 June 2017); (**b**–**d**) *Tychonema* sp. filaments within water moss clumps (b: 2 June 2017; c-d: 6 June 2017; b: ×100; c: ×400, d: ×1000); (**e**) *Tychonema* sp. filament observed in the stomach contents of a dead dog (×200).

Histological examinations revealed acute necrosis of renal tubular epithelial cells in both dogs. In the gastric lumen, filaments resembling *Tychonema* sp. were observed (Figure 1e). Importantly, no hepatic lesions with clinical significance were detected in the liver of both dogs. Incidental findings included a small hepatic lipogranuloma (dog D3) and minimal pyogranulomatous pneumonia were diagnosed (dog D1), however, of no clinical relevance. Furthermore, the spleen of both dogs showed a lymphatic depletion.

### 2.2. Macrophyte and Phytoplankton Samples

Near the bathing sites and lake shores where the dog intoxications occurred, large areas with water moss (*Fontinalis antipyretica*) floating at the water surface were observed (Figure 1a). Microscopic investigation of the water moss revealed the presence of filamentous cyanobacteria, which were identified as *Tychonema* sp. based on morphological traits (Figure 1b–d) [22]. The cells were isodiametric with filament width varying between 6.5 and 8.9 µm on 6 June 2017. As filament width is one criterion for species differentiation in *Tychonema*, species identification was not unequivocally possible. *Tychonema* sp. was found with a biovolume of 24.4 mm^3^ L^−1^ and an abundance of 67 × 10^6^ cells per liter in water from *Fontinalis* clumps on 6 June 2017.

The qualitative investigation of the littoral samples near the bathing site showed a biocoenosis typical of littoral sandy habitats [23], dominated by diatoms, but also with *Tychonema* sp. not been observed at this site before. Biovolumes of *Tychonema* sp. in the water samples from 30 May and 6 June 2017 were 0.4 and 0.1 mm^3^ L^−1^, respectively.

Filaments identical to *Tychonema* sp. could also be found in the stomach contents of the dogs (Figure 1e).

### 2.3. Cyanobacterial and Other Toxins

**Figure 2:**
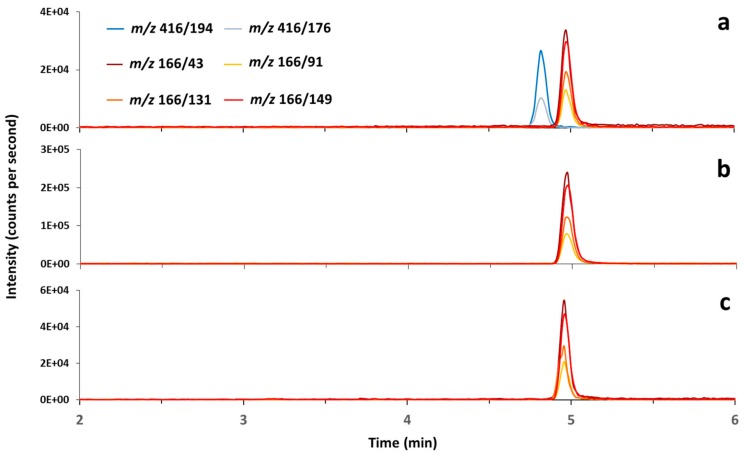
Reconstructed LC-MS/MS chromatograms of (**a**) certified reference standards; (**b**) the aqueous fraction from *Fontinalis*/*Tychonema* clumps from Reiherwerder, 6 June 2017 (WT1, Table 2); (**c**) the stomach contents of dog D1 (Table 2). The blue lines show the transitions indicative for cylindrospermopsin; red, orange and yellow lines show transitions for anatoxin-a.

ATX was clearly detected in the *Fontinalis*/*Tychonema* clumps and the stomach contents of the dogs (Figure 2). Two peaks with fragment ions indicative of dihydroanatoxin-a (DATX) were found in WT1; however, the ratios of the ions were inverse between the peaks (data not shown). Thus, the presence of DATX could not be unequivocally verified.

Neither microcystins and cylindrospermopsin, nor homoanatoxin-a and its analogues were detected in the *Fontinalis*/*Tychonema* clumps and stomach contents of the dogs. No other organic toxins such as rodenticides, organophosphorus insecticides, carbamates, neonicotinoids, metaldehyde and chlorinated hydrocarbons were detected.

The ATX concentrations in the aqueous fraction of two *Fontinalis*/*Tychonema* clumps sampled on 6 June 2017 near the beach where the dog casualties occurred amounted to 943 and 1870 µg L^−1^, respectively (FT1, Table 2). *Fontinalis* sampled at other sites in Lake Tegel or at later time points showed much lower, though still considerable, ATX concentrations (FT2–4, Table 2). No ATX or only traces of ATX were found in sand and the open water of different sites near *Fontinalis* accumulations (S1, W1–3, Table 2). 

In the stomach contents of dogs D1 and D3, ATX concentrations of 8700 and 7320 µg L^−1^ were detected, respectively (Table 2). Around 6 µg L^−1^ ATX was found in the stomach contents of dog D2, which received, in contrast to the other two dogs, a gastric lavage as initial treatment (Table 2).

Two screening campaigns conducted in the second half of June 2017 and in August/September 2017 in Lake Tegel and other lakes in Berlin showed only very occasional traces of ATX at concentrations <1 µg L^−1^ both in macrophytes and pelagic water samples (data not shown).

## 3. Discussion

The massive occurrence of *Tychonema* associated with *Fontinalis* has neither been described in the literature nor observed before in water bodies around Berlin, and the intoxication of dogs reported here is the first observation of its kind in the wider region. Circumstances were thus completely unanticipated and unfamiliar, sampling was partly not performed systematically and is therefore not comprehensive. However, through close communication between the agencies and institutions involved, a variety of samples were collected in a narrow timeframe after the first incident came to our attention. As a result, the dataset is inevitably incomplete with respect to the dynamics of *Tychonema* abundance and toxicity, the role of *Fontinalis* and other aspects. Nonetheless, the collected data provide strong evidence that the death of at least some of the dogs was caused by ATX produced by *Tychonema* sp., as discussed in the following text.

The fast clinical onset as well as the symptoms of neurotoxicosis observed in the dog casualties are in line with previous reports of ATX intoxication in dogs [10,11,13]. ATX was the only toxin identified in the stomachs of all analyzed dogs, whereas other (neuro)toxins were not detected. The pathological examination of the two dogs revealed no common lesions other than hemorrhages and acute renal tubular cell necrosis. While nephrotoxicity has been observed for the cyanotoxins microcystin or nodularin after intraperitoneal administration, this has not been reported for ATX so far [1]. Acute renal necrosis and hemorrhages often occur following any kind of shock and are not the result of a direct effect of the toxin. Thus, these lesions must be interpreted as nonspecific. Hepatocellular necrosis, which can be caused by cyanobacterial toxins such as microcystin, was not diagnosed in the investigated dogs, reflecting the absence of these cyanobacterial hepatotoxins in the *Fontinalis*/*Tychonema* clumps and stomach samples. Splenic lymphoid depletion had been reported in dogs following nodularin intoxication [24]. In contrast to the cases in this study, depletion of lymphocytes had also been observed in other lymphoid organs such as the lymphnode and thymus [24]. ATX is not known to be immunotoxic and is unlikely to be the cause of the observed splenic lymphoid depletion. Such a lesion is a common incidental finding seen in many dogs and is unrelated to intoxication. Taken together, these facts underline the high probability of ATX intoxication as the cause of death in the two investigated dogs and it is tempting to assume that the other dogs with symptoms of neurotoxicosis may also have suffered from ATX poisoning.

The analysis of ATX from natural specimen by LC-MS is challenging and misidentifications have occurred in the past [25]. The reason is that ATX has the same molecular weight and some identical product ions as the amino acid phenylalanine as well as a similar chromatographic behavior [25,26]. In this study, ATX was clearly separated from phenylalanine and four different transitions for ATX have been monitored to ensure an unequivocal identification of ATX [25]. The detection of HATX and its analogues as well as ATX analogues in this study relied on monitoring of published product ions, as no reference material was available [13,26]. Two peaks, one eluting shortly before, the other co-eluting with ATX, both showed the transitions indicative of dihydroanatoxin-a, however in inverse ratios. As no reference material is available, further mass spectrometric investigations (i.e., MS^3^) are needed for ultimate confirmation [26]. 

Due to its high sensitivity, modern MS/MS instrumentation enables the direct analysis of water or crude extracts without preceding concentration steps, e.g., by solid phase extraction (SPE). However, SPE may also assist in sample clean-up and thus help to reduce matrices possibly leading to ion suppression or enhancement in LC-MS/MS [27]. No SPE has been applied in this study as the concentration of ATX was sufficient for direct analysis. On the contrary, samples from *Fontinalis*/*Tychonema* clumps and the stomach contents of the dogs had to be diluted between 100–1000-fold prior to analysis. Dilution in this range has been shown to greatly reduce the influence of even complex matrices, thus enabling robust quantification [28]. 

In the absence of cyanobacterial surface blooms in Lake Tegel, several other potential causes (i.e., toxins) for the observed neurotoxicosis had been explored before sampling of water moss at the site where the intoxication occurred revealed the presence of filamentous cyanobacteria. The cyanobacterium was identified as *Tychonema* sp. based on morphological traits [22] and was found primarily associated to *Fontinalis*, though it is not known if the filaments proliferated massively already in the usual growing depth of *Fontinalis* in Lake Tegel (ca. 1–4 m, [21]) or only in detached and floating *Fontinalis*. As *Tychonema* sp. has also been observed in the plankton outside the *Fontinalis* clumps, it thus can be characterized as tychoplanktic, i.e., being neither attached nor being truly planktic and present among macrophytes and in the litoral zone [29]. This is characteristic for two species of *Tychonema*, *T. tenue* and *T. bornetii*, of which *T. tenue* has narrower trichomes (5–7 µm) than *T. bornettii* (8/12–16 µm) [22]. As trichome width ranged from 6.8–8.9 µm in *Tychonema* from Lake Tegel, unequivocal species identification was not possible based on morphological characteristics alone. Genetic characterization of isolated strains may support species identification [30,31], but attempts to establish cultures were futile.

*Tychonema* sp. strains from different countries have been identified as ATX producers based on the detection of the ATX encoding genes (*ana*) and LC-MS/MS analysis [30]. Cell quota of ATX in *Tychonema* strains were in the range of 0.1 and 2.6 µg mm^−3^ biovolume and 0.01 and 0.35 pg cell^−1^, which is comparable to the ATX cell quota in other ATX producing species as well as for other cyanotoxins such as cylindrospermopsin and microcystin [30,32,33,34]. In addition to ATX, Italian strains of *T. bourrellyi* produce minor amounts of homoanatoxin-a [35], which has not been detected in Lake Tegel to date. However, there were indications of the presence of dihydroanatoxin-a in the *Fontinalis*/*Tychonema* samples from Lake Tegel, which is interesting, as only recently dihydroanatoxin-a has been shown to be not only an ATX degradation product, but also an ATX analogue synthesised by *Cylindrospermum stagnale* [26,36].

Concentrations of ATX in North Italian lakes (Garda, Como, Iseo, and Maggiore) associated with planktic *T. bourrellyi* were usually below 4 µg L^−1^ [35]. In contrast, ATX concentrations in *Fontinalis*/*Tychonema* clumps from Lake Tegel were much higher due to the high biomass of *Tychonema* within the water moss. Considering the high concentrations of ATX in the stomach contents of the dogs of around 8000 µg L^−1^, it is probable that ATX concentrations in the *Fontinalis*/*Tychonema* clumps were even higher at the time when the dog casualties occurred. No ATX or considerably lower concentrations of ATX were detected in the open water or in *Fontinalis* samples from other parts of the lake underlining the patchiness of the association of toxigenic *Tychonema* with *Fontinalis*. Screening campaigns later in the year very rarely revealed traces of ATX <1 µg L^−1^ in *Fontinalis* or the open water, indicating a higher abundance of *Tychonema* only in spring, which has also been reported for *T. bourrellyi* from the North Italian lakes [35].

The recent occurrence of planktic, ATX-producing *T. bourrellyi* in North Italian lakes has been discussed in relation to their re-oligotrophication [35], and this is also the situation of Lake Tegel. However, *Tychonema* sp. observed in Lake Tegel was tychoplanktic and thus it remains to be investigated under which conditions *Tychonema* sp. can proliferate in stands of *Fontinalis*. Multiple scenarios ranging from a unique event under very specific conditions to future regular occurrences in Lake Tegel are conceivable. This emphasizes the specific challenges associated with the development of appropriate surveillance schemes for tychoplanktic cyanobacteria: the extent to which they can draw on schemes for planktic or benthic cyanobacteria is limited. This is particularly relevant for lakes for which restoration measures have been successful and most water quality parameters such as transparency have significantly improved.

Further investigations on the occurrence of potentially toxic, non-bloom forming cyanobacteria in less eutrophic water bodies are needed for a better understanding of this potential health hazard. As initial measures to mitigate human and animal health risks, bathers and pet owners should be advised to minimize the ingestion of lake water and to avoid the uptake of water plants, even in the absence of algal blooms and in apparently clear waters. Furthermore, the occurrence of floating macrophytes in water bodies such as Lake Tegel needs to be monitored during bathing site inspection. A protective measure includes their mechanical removal, as was performed in summer 2017 when their role in the dog casualties had been discovered.

## 4. Materials and Methods

### 4.1. Study Site

Lake Tegel is situated in the northwest of Berlin, Germany (52.576111 N 13.25333 E). With a surface area of 3 km^2^, it is one of the largest lakes within the city. The high share of sewage in its two major contributors led to hypertrophic conditions with recurrent and intense cyanobacterial blooms during the 1970s [18]. As it serves as a major drinking water source via bank filtration as well as for recreation, its restoration started in 1984 by phosphorus removal in its main inflow [18]. The lake recovered gradually and now displays mesotrophic conditions [20]. 

### 4.2. Animal Necroscopy

Two dogs (Table 1) were submitted for a complete postmortem examination. Tissue samples of liver, kidney, brain, stomach, small and large intestine, lung, heart, pancreas, tonsil, bone marrow, thymus, pancreas, spleen, lymph node, brain, thyroid and adrenal glands were immersion-fixed in 4% buffered formalin, dehydrated and embedded in paraffin wax. Five micrometers of hematoxylin and eosin stained tissue sections were histopathologically analyzed. Additionally, tissues of liver, kidney, brain, skeletal muscle, adipose tissue, stomach, small and large intestine, as well as the content of the gastrointestinal tract, blood and bile were stored at −20 °C. Although a full necropsy of dog D2, which was submitted deep-frozen, was not performed, its stomach contents were sampled for further analysis.

### 4.3. Macrophyte, Phytoplankton and Toxin Samples

Water samples for toxin analysis and/or phytoplankton analysis were taken near the surface at 50 cm water depth, opposite the isle Reiherwerder at the official bathing site (52.585502 N 13.255432 E) on 30 May and 6 June 2017 in close vicinity to the places where dog casualties were reported. Additionally, clumps of water moss were collected within the littoral on 6 June. During the following summer season, water, sediment (sand) and water moss were repeatedly taken from different bathing sites at Lake Tegel and other lakes in Berlin.

Water and moss samples for toxin analysis were immediately frozen at −20 °C. Phytoplankton samples were preserved with Lugol’s solution. Cell densities and biovolumes were estimated according to DIN EN 15204 [37] in sedimentation chambers at different magnifications (200× and 400×) using an inverted microscope (Olympus IX71). Unpreserved water obtained from sampled moss clumps was examined qualitatively for the presence and taxonomic determination of cyanobacteria. Additionally, the abundance of *Tychonema* sp. was estimated by counting the biovolume in an aqueous fraction of *Fontinalis*.

### 4.4. Toxin Analysis

#### 4.4.1. Cyanotoxins

For the analysis of ATX and CYN, samples of water, water moss and the stomach contents of the dogs were frozen/thawed twice and acidified with formic acid to a final concentration of 0.1% formic acid (FA). Samples for MC analysis were diluted with 75% MeOH to a final volume of 75% aqueous methanol [38]. All samples were ultrasonicated for 10 min, shaken for 1 h, centrifuged and filtered (0.2 µm, PVDF, Whatman, Maidstone, UK) before analysis by LC-MS/MS.

LC-MS analysis was carried out on an Agilent 2900 series HPLC system (Agilent Technologies, Waldbronn, Germany) coupled to a API 5500 QTrap mass spectrometer (AB Sciex, Framingham, MA, USA) equipped with a turbo-ionspray interface. 

For MC analysis, the extracts were separated using a Purospher STAR RP-18 endcapped column (30 mm, 4 mm, 3 mm particle size, Merck, Germany) at 30 °C [39]. The mobile phase consisted of 0.5% FA (A) and acetonitrile with 0.5% FA (B) at a flow rate of 0.5 mL min^−1^ with the following gradient program: 0 min 25% B, 10 min 70% B, 11 min 70% B. The injection volume was 10 µL. Identification of MCs was performed in the positive Multiple Reaction Monitoring (MRM) mode with the transitions and mass spectrometric parameters given in Table S1 (Supplementary Materials). [Asp^3^]-MC-RR, MC-YR, [Asp^3^]-MC-LR, MC-LR, MC-LW, MC-LF, MC-LA were from Enzo Life Sciences (Lörrach, Germany), MC-RR and MC-LR from National Research Council (Ottawa, ON, Canada). The detection limits of different congeners ranged from 0.03 to 0.5 µg L^−1^.

For the LC–MS/MS analysis of CYN and ATX-a, the extracts were separated using a 5 mm Atlantis C18 (2.1 mm, 150 mm column, Waters, Eschborn, Germany) at 30 °C. The mobile phase consisted of water (A) and methanol (B) both containing 0.1% FA, and was delivered as a linear gradient from 1% to 25% B within 5 min at a flow rate of 0.25 mL min^−1^. The injection volume was 10 µL. Identification of CYN and ATX was performed in the positive MRM mode with the following transitions: CYN *m*/*z* 416.1 [M + H]^+^ to 194 and 416.1/176, and ATX *m*/*z* 166.1 [M + H]^+^ to 149, 166.1/131, 166.1/91, 166.1/43. The detection limit was 0.02 µg L^−1^ for CYN and ATX. In addition, homoanatoxin-a (HATX) and its analogues dihydrohomoanatoxin-a (DHATX) and epoxyhomoanatoxin-a (EHATX) as well as the ATX analogues dihydroanatoxin-a (DATX) and epoxyanatoxin-a (EATX) were monitored using fragment ions described in the literature as no reference material was available [13,26]. Transitions monitored and mass spectrometric parameters are given in Table S1 (Supplementary Materials).

#### 4.4.2. Other Toxins

The stomach contents of dog D1 were further analyzed for organic compounds including rodenticides, organophosphorus insecticides, carbamates, neonicotinoids, metaldehyde and chlorinated hydrocarbons by gas chromatographic mass spectrometry (GC/MS).

Briefly, stomach contents were extracted with a mixture of diethyl ether and ethyl acetate (1:1, *v*/*v*). Part of the extract was treated with trimethylsulfonium hydroxide (TMSH) to form methyl derivatives [40]. Analyses were performed by gas chromatography–mass spectrometry in full-scan electron ionization (EI) mode. All compounds were identified by matching their mass spectra with spectral libraries and by chemical fragment structure interpretation [40].

Further, the stomach contents of dogs D1 and D3 were analyzed for the presence of rodenticides (zinc phosphide, aluminium phosphide). The determination of phosphide was performed by a Dräger detection tubes system and Dräger accuro gas detection pump (Dräger Safety, Lübeck, Germany). Analyses of zinc phosphide or aluminium phospide are based on the detection of liberated phosphine gas from stomach contents by a Dräger phosphine 0.01/a tube [41]. The detection limit is 0.01 ppm phosphine.

## Figures and Tables

**Table 1:** Summary of dogs with neurotoxicosis analyzed here.

No.	Breed (Sex)	Body Weight (kg)	Age (Years)	Case History and Symptoms	Treatment	Outcome
D1	American Bulldog (Female)	38.5	2.5	Swimming in Lake Tegel, sudden onset of neurological failures of hind limbs, collapse, dyspnea and cyanosis	No treatment	Death
D2	Labrador half-breed (Female)	No data	1	Swimming in Lake Tegel, sudden onset of paralysis of all limbs, foam at mouth, apnea	Gastric lavage with sodium bicarbonate, doxapram hydrochloride intravenous	Euthanized
D3	French Bulldog (Female)	8.8	0.9	Playing at Lake Tegel, sudden onset of paralysis of all limbs, foam at mouth, apathy, dyspnea	No treatment	Death

**Table 2:** ATX concentrations in different specimens and water samples (µg/L).

Code	Sample	Organ/Geographical Site	Sampling Date	ATX (µg L^−1^)
D1	Dog 1 S270/17	Stomach contents		8700
D2	Dog 2 S298/17	Stomach contents		5.7
D3	Dog 3 S271/17	Stomach contents		7320
FT1	*Fontinalis*/*Tychonema*	L. Tegel, Reiherwerder	6 June 2017 (1)	943
			6 June 2017 (2)	1870
FT2	*Fontinalis*/*Tychonema*	L. Tegel, Scharfenberg	6 June 2017	19.8
FT3	*Fontinalis*/*Tychonema*	L. Tegel, Reiswerder	10 June 2017 (1)	24.4
			10 June 2017 (2)	12.6
FT4	*Fontinalis*/*Tychonema*	L. Tegel, Reiherwerder (2 m)	19 June 2017	<LOQ
		L. Tegel, Reiherwerder (4 m)		0.4
S1	Sand	L. Tegel, Reiswerder	10 June 2017	1.0
W1	Pelagic water	L. Tegel, Reiherwerder	6 June 2017	n.d.
W2	Pelagic water	L. Tegel, Reiherwerder	30 May 2017	1.0
W3	Pelagic water	L. Tegel, Reiswerder	10 June 2017	n.d.

n.d. not detected; LOQ: Limit of Quantification.

## Supplementary Materials

The following table is available online at http://www.mdpi.com/2072-6651/10/2/60/s1, Table S1: Mass spectrometer parameters for the analytes monitored.
