# Supplementary material for: Fatal Neurotoxicosis in Dogs Associated with Tychoplanktic, Anatoxin-a Producing Tychonema sp. in Mesotrophic Lake Tegel, Berlin
Source: Toxins (Basel). 2018 Jan 31;10(2):60. doi: 10.3390/toxins10020060 (PMC5848161; doi:10.3390/toxins10020060)
Supplement: Supplementary file 1 [file toxins-10-00060-s001.pdf]

Supplementary Materials: Fatal Neurotoxicosis in Dogs Associated with Tychoplanktic, Anatoxin-a Producing *Tychonema* sp. in Mesotrophic Lake Tegel, Berlin

Jutta Fastner, Camilla Beulker, Britta Geiser, Anja Hoffmann, Roswitha Kröger , Kinga Teske, Judith Hoppe, Lars Mundhenk, Hartmud Neurath, Daniel Sagebiel and Ingrid Chorus

Table S1. Mass spectrometer parameters for the analytes monitored.

Analyte	MRM transitions monitored (*m/z*)	DP (V)	CE (V)
Anatoxin-a	166.1 > 149	51	19
	166.1 > 131		23
	166.1 > 91		31
	166.1 > 43		45
Homoanatoxin-a	180.1 > 163	51	25
	180.1 > 145		25
Dihydroanatoxin-a	168.0 > 150	51	25
	168.0 > 133		25
Epoxyanatoxin-a	182.0 > 164	51	25
	182.0 > 138		25
Dihydrohomoanatoxin-a	182.0 > 164	51	25
	182.0 > 147		25
Epoxyhomoanatoxin-a	196.0 > 178	51	25
	196.0 > 138		25
Cylindrospermopsin	416.1 > 194	105	48
	416.1 > 176		48
MC-LR	995.5 > 135	236	123
	995.5 > 213		75
MC-RR	519.7 > 135	121	42
	519.7 > 70		129
MC-YR	1045.5 > 135	121	129
	1045.5 > 213		75
[Asp^3]-MC-RR	512.8 > 135	141	35
	512.8 > 213		45
[Asp^3]-MC-LR	981.5 > 135	231	113
	981.5 > 213		73
MC-LF	986.5 > 135	166	97
	986.5 > 135		71
MC-LW	1025.5 > 135	176	101
	1025.5 > 213		83
MC-LA	910.5 > 135	31	95
	910.5 > 213		69

DP: declustering potential, CE: collision energy.
